# Methylation in hangtaimycin biosynthesis and its antibacterial activities

**DOI:** 10.1016/j.synbio.2023.10.003

**Published:** 2023-10-30

**Authors:** Minghe Luo, Yulu Dong, Zixin Deng, Yuhui Sun

**Affiliations:** a School of Pharmacy and Bioengineering, Chongqing University of Technology, Chongqing, China; b Key Laboratory of Combinatorial Biosynthesis and Drug Discovery, Ministry of Education, and School of Pharmaceutical Sciences, Wuhan University, Wuhan, China

**Keywords:** Hangtaimycin, Methylation, *S*-Adenosylmethionine-dependent methyltransferases, FK506 methyltransferase type *O*-methyltransferase, Antibacterial, Biosynthesis

## Abstract

About two-thirds of small molecule drugs contain methyl group and it plays a very important role in the drug development. So, methyltransferases catalyzing the methylation have always attracted great attention. Hangtaimycin (HTM) is a potent hepatoprotective agent. Previous study showed that its biosynthetic gene cluster contained three methyltransferase domains, but their characteristics in HTM biosynthetic pathway has not been revealed. In this study, we clarified multi-methylations in HTM biosynthesis in vivo. It showed that the two *S*-adenosylmethionine-dependent methyltransferases (SAM-MTs) of HtmA2(-module 6)-MT domain and HtmB2(-module 18)-MT domain are responsible for the installation of methyl group at C-45 and N-12, respectively, whereas the FK506 methyltransferase (FKMT) type *O*-methyltransferase of HtmB1(-module 16)-MT domain take care of the methylation at O-21 of HTM. We also reported the antibacterial activities of HTM in this study, and found that it showed activities against *M*. *luteus*, *B. thuringiensis* and *A. baumannii* with MIC of 4 μg/mL, 4 μg/mL, and 64 μg/mL, respectively.

## 1 Introduction

The methyl group, one of the most commonly occurring carbon fragments, appears in nearly 67% of the top-selling small-molecule drugs and can modulate both the biological and physical properties of drugs [1]. The abilities of methylation to optimize properties of candidate drugs, highlights its important role in drug discovery [2]. For example, the methylation in simvastatin could sterically block metabolism and lengthen its half-life [3,4]. Methylation also were found to have favorable effects on solubility [5] or selectivity against off-targets [6]. One reason for the population of methyl group in drug development is its magic methyl effect: installation of a methyl group could induce a conformational change giving rise up to 590-fold boosts in potency, for example the outcome of analogue ML298-9g of compound ML298 soar significantly, compared with that of ML298-9f [1,7]. The diversity role of methylation is determined by the wildly distributed methyltransferase in nature to catalyze the methylation reaction [8]. There are many classes of methyltransferase [[9], [10], [11]], such as *S*-adenosylmethionine-dependent methyltransferases (SAM-MTs) and FK506 methyltransferase (FKMT) type *O*-methyltransferase, which has a character of lacking the binding site conserved motif of “E/D × G × G × G” of SAM-MTs and unknown of its clear catalytic mechanism [12]. The majority of methylation reactions are catalyzed by SAM-MTs [9].

**Fig. 1:**
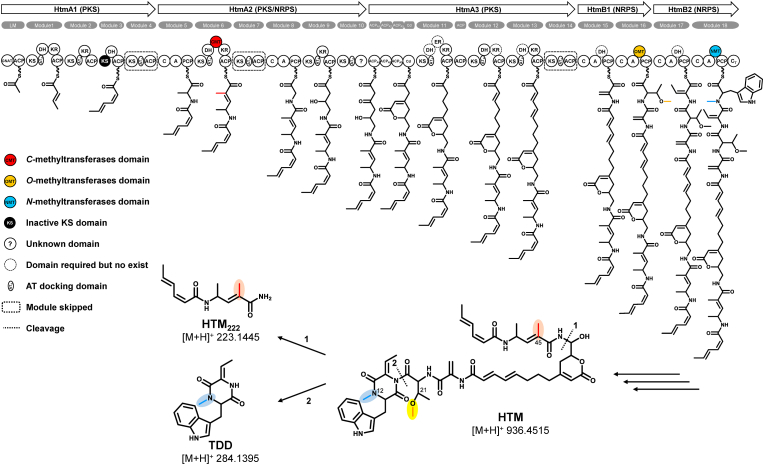
Proposed biosynthetic pathway for HTM, HTM_222_ and TDD. The methyl groups and corresponding methyltransferase domains involved in this study are highlighted in colors.

Hangtaimycin (HTM), a product of a hybrid polyketide synthase/nonribosomal peptide synthetase (PKS/NRPS), containing a rare unsaturated hexalactone ring embedded in a conjugated dodecanoic acid and a terminal diketpiperazine units (Fig. 1). Accompany with the unique structure of HTM, it presents good hepatoprotective bioactivities [13]. Previous work showed that, during its biosynthesis, there existed dehydrating bimodules to install a double bond in HtmA1 [12] and a P450 enzyme to add a hydroxyl group at C-42 [14], but the exact methylation in its biosynthetic pathway is still unknown. In this study we report the characterization of the three methylases in HTM biosynthesis. We also report the HTM's antibacterial activities against *M*. *luteus*, *B. thuringiensis* and *A. baumannii*.

## 2 Materials and methods

### 2.1 Bacterial strains, plasmids and DNA manipulation

Bacterial strains and plasmids of this study are listed in Table S1 of Supporting Information. DNA manipulations were operated by standard procedures for *E. coli* and *Streptomyces*. All chemical reagents and antibiotics were obtained from Sigma-Aldrich. Primers were synthesized by Tsingke (Supporting Information, Table S2). DNA sequencing of PCR products was performed by Tsingke.

### 2.2 Culture and fermentation conditions

The wild-type and mutants of *Streptomyces spectabilis* CCTCC M2017417 were grown on ABB13 plates (0.5% soytone, 0.5% soluble starch, 0.3% CaCO_3_, 0.2% MOPS, 2% agar). The seed culture was obtained in TSBY liquid medium (3% tryptone soy broth, 10.3% sucrose, 0.5% yeast extract) at 28 °C with shaking of 220 rpm for 20 h. And then, the seed culture was inoculated to the modified SFM medium (2% mannitol, 2% soya flour, 5% AB-8 macroporous adsorption resin) and fermented for another 4 days at 28 °C and 220 rpm. *E. coli* strains were cultured in 2 × TY (1.6% tryptone, 1% yeast extract, 0.5% NaCl) or on 2 × TY agar medium at 37 °C with the appropriate antibiotic at a concentration of 100 μg/mL ampicillin, 35 μg/mL apramycin, or 30 μg/mL nalidixic acid for selection.

### 2.3 Domain or gene disruption in vivo

The constructs used for gene disruption were introduced into *S. spectabilis* CCTCC M2017417 by conjugation using donor strain ET12567/pUZ8002 on ABB13 plates. After incubation at 28 °C for 12 h, the plate was overlaid with the final concentration of 35 μg/mL apramycin and 30 μg/mL nalidixic acid. Exconjugants were selected on ABB13 plates supplied with 35 μg/mL apramycin and 30 μg/mL nalidixic acid to confirm their antibiotic resistance. Then single colonies were patched onto ABB13 plates containing 35 μg/mL apramycin and onto ABB13 plates without antibiotic, respectively, to screen for the double crossover mutant. The correct phenotype (Apr^R^) candidate mutants were further verified by PCR and sequencing.

### 2.4 Construction of in-frame gene disruption plasmid

To obtain the HtmB2(-module 18)-MT domain in-frame deletion mutant, two homologous recombination fragments were amplified by PCR using primer pairs of OMT-L-up and OMT-L-re, OMT-R-up and OMT-R-re, respectively (Supporting Information, Table S2). After digesting and recycling of the Streptomyces-E. coli shuttle vectors pYH7 with NdeI and HindIII, the two homologous fragments of HtmOMT were cloned into pYH7 by three-piece ligation of Gibson method to create the disruption plasmid pWHU-OMT. The primers used to verify the in-frame deletion in the plasmid and the mutants were list in Table S2 of Supporting Information.

### 2.5 Construction of site-directed mutation plasmids

To construct HtmCMT and HtmNMT site-directed mutation plasmids, each two homologous recombination fragments were amplified from the genome using primer pairs listed in Table S2 of Supporting Information and fused into the vector pYH7 digested with NdeI and HindIII to yield the site-directed mutation plasmids of pWHU-CMT and pWHU-NMT, respectively. The primers used to verify the site-directed mutation in the plasmid and the mutants were list in Table S2 of Supporting Information.

### 2.6 Degradation of HTM in vitro

HTM which was isolated by the method mentioned in our previous work [13] with a yield of nearly 6.5 mg/L was dissolved in acetonitrile and then left at room temperature for 0−9 days. At the day of 0, 3, 6 and 9 days, the dissolved HTM in acetonitrile was analyzed by LC-ESI-HRMS (Liquid Chromatography Electrospray Ionization High Resolution Mass Spectrometry) to detect the content variation of HTM, HTM_222_ and TDD, respectively. The LC-ESI-HRMS analytical method showed below.

### 2.7 HPLC and LC-ESI-HRMS analysis of the extracts of wild-type and mutant strains

After fermentation in modified SFM medium [13] for 4 days, the cultures of wild-type and mutant strains of *S. spectabilis* CCTCC M2017417 were extracted with ethyl acetate. After the evaporation of ethyl acetate under reduced pressure, the extracts were dissolved in 1.5 mL acetonitrile and analyzed by HPLC at 264 nm with a Cosmosil 5C4-AR-300 Packed column (250 × 4.6 mm, 5 μm) eluting with a linear gradient elution system of CH_3_CN/H_2_O (0−20 min 35:65–65:35; 20−25 min 65:35–100:0; 25−30 min 100:0; 30−35 min 35:65) at a flow rate of 1 mL/min, on an equipment of Shimadzu LC-20A. Meanwhile LC-ESI-HRMS was carried out on a Thermo Electron LTQ-Orbitrap XL mass spectrometer in a positive ionization mode with 35% relative collision energy, equipted with a linear gradient elution system of CH_3_CN/H_2_O (0–20 min, 35–65% B; 20–25 min, 35% B) at a flow rate of 1 mL/min.

### 2.8 Antibacterial activities

HTM was further tested for its antibacterial activities under the method described before [15]. Briefly, the tested compounds were firstly dissolved individually in DMSO as mother liquor, which were serially diluted to various concentrations in each cells. Then the tested eight strains of *Micrococcus luteus* ATCC 10240, *Bacillus thuringiensis* BT01, *Bacillus subtilis* CGMCC 1.3358, *Staphylococcus aureus* ATCC 29213, *Acinetobacter baumannii* ATCC 19606, *Klebsiella pneumoniae* ATCC 13883, *Escherichia coli* ATCC 25922, and *Pseudomonas aeruginosa* ATCC 47085 were added to each cell with a final concentration of 10^6^ CFU/mL, respectively. After incubation at 37 °C for 16–20 h, the compound's concentration in cells with no turbid were defined as the MIC of the tested compounds, respectively. Experiments are triplicated for three times. Apramycin and kanamycin served as the positive controls, while DMSO served as the negative control.

## 3 Results

### 3.1 Formation of HTM derivatives of HTM_222_ and TDD

**Fig. 2:**
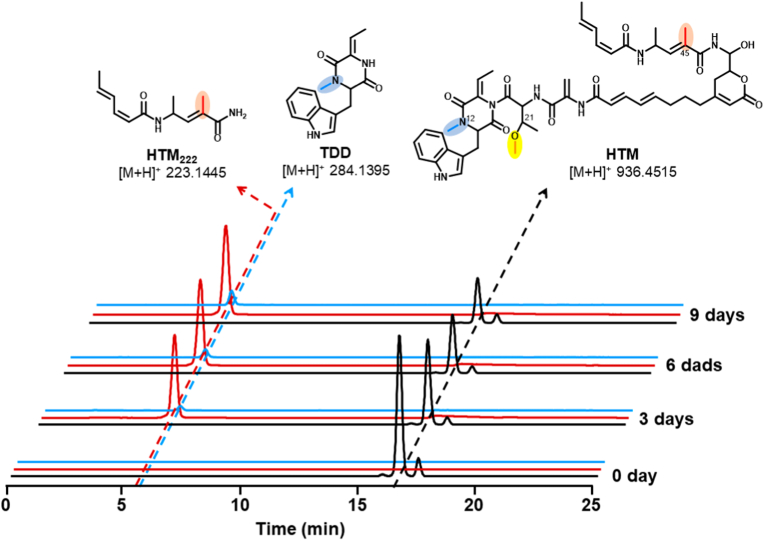
LC-ESI-HRMS analysis of degradation of HTM to TDD and HTM_222_.

As has been demonstrated, a cytochrome P450 monooxygenase in HTM biosynthesis is responsible for the C-42 oxidation of deoxyhangtaimycin to HTM [14]. The installed hemiaminal functionality is unstable and easily lead to the degradation of HTM into HTM_222_ and a larger lactone-polyene peptide fragment [16]. Another HTM cometabolite in *S. spectabilis* is tryptophan-dehydrobutyrine diketopiperazine (TDD), which had been isolated for several decades ahead of HTM, but its absolute configuration was confused [[17], [18], [19]]. In HTM biosynthesis, HTM and TDD was found to be simultaneous disappearance and appearance, suggesting the same set of biosynthetic machinery for HTM [16,19]. The formation mechanism of TDD is still unknown, in our opinion, it may be formed by an unidentified enzyme in HTM's biosynthesis. Recently, Xu et al. had established the *S* configuration for the natural product TDD from *S. spectabilis* by comparing with synthetic standard, which is likely reflected in the corresponding portion of HTM, for the reason of absence of *E* domain in HTM biosynthesis [16]. To further confirm that the formation of TDD and HTM_222_ could be part of the cause of the degradation of HTM (Fig. 1). We conducted HTM degradation experiment in vitro by time-course of 0, 3, 6 and 9 days, respectively. Results showed that the content of HTM gradually decreased with time, while the content of HTM_222_ and TDD increased accordingly (Fig. 2), indicating that at least part of HTM_222_ and TDD must be produced by HTM degradation.

### 3.2 *C*, *N*-methylation in HTM biosynthesis

**Fig. 3:**
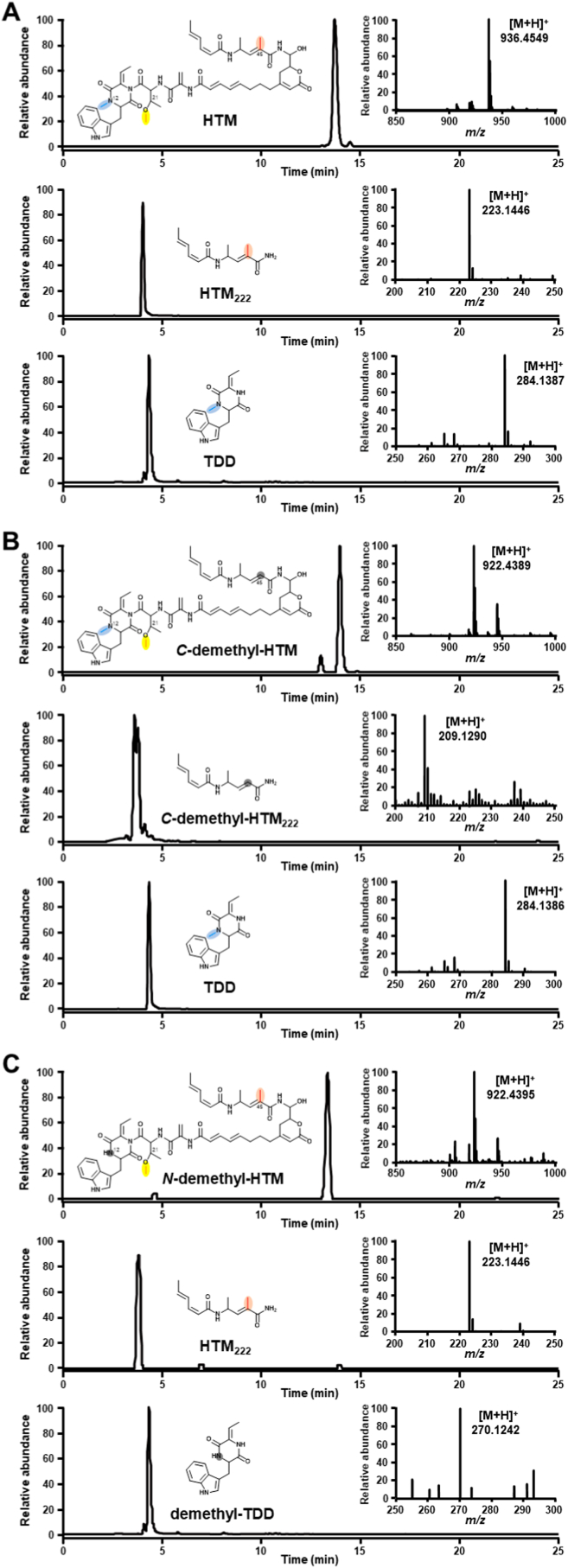
LC-ESI-HRMS analysis of the fermentation extracts of wild-type (A), CMT(G132D, G134W, G136D) (B) and NTM(G108R, G110R, G112R) (C).

According to the structure and the biosynthetic gene cluster analysis of HTM, three methyltransferase domains, including two SAM-MTs and a FKMT type *O*-methyltransferase [13] are existed in module 6, 16 and 18 of PKS/NRPS, which are supposed to catalyze the three methylations at the three methylation sites in HTM structure (Fig. 1). In order to identify the exact methylation sites of these three methyltransferase domains, the in vivo genetic inactivations were conducted. Since the SAM-MTs have conserved motif of “E/D × G × G × G” [8], the site-directed inactivation of the SAM-MTs of *C*-methylase (D × G × G × G″ was mutated to D × **D** × **W** × **D**) and *N*-methylase (E × G × G × G was mutated to E × **R** × **R** × **R**) in HTM biosynthetic gene cluster were performed, respectively (Fig. S1−S3). HPLC-DAD analytical results showed that the production of HTM was abolished in both the mutants of CMT(G132D, G134W, G136D) and NTM(G108R, G110R, G112R) (Fig. S4), and as expected, HTM derivatives (demethyl-HTM) with the remove of only one methyl in HTM can also be extracted in high resolution mass spectrometry (Fig. 3). Considering the hemiaminal in HTM could lead to the slowly decomposition to corresponding aldehyde and the amine (HTM_222_) [13] (Fig. 2), we think this feature and the fact that TDD and HTM existing concurrently [19] would facilitate to identify which methyltransferase domain is responded for the corresponding site in HTM structure. In both mutants of CMT(G132D, G134W, G136D) and NTM(G108R, G110R, G112R), not only the demethyl-HTM can be detected precisely, but also the mass spectrometry of demethyl-HTM_222_ and demethyl-TDD, which are corresponding to the fragment of *C*-demethyl-HTM and *N*-demethyl-HTM were found from mutants of CMT(G132D, G134W, G136D) and NTM(G108R, G110R, G112R), respectively (Fig. 3). These results suggested that HtmA2(-module 6)-MT domain is responsible for the methylation at C-45 and HtmB2(-module 18)-MT domain is responsible for the methylation at N-12 in HTM.

### 3.3 *O*-methylation in HTM biosynthesis

After determination of the two SAM-MTs of *C-* and *N*-methylation at C-45 and N-12 in HTM, the remaining methylation at O-21 in HTM may be catalyzed by the FKMT type *O*-methyltransferase domain in module 16 of NRPS HtmB1. It is interesting that a FkbM family methyltransferase motif of “DxGxNxG” corresponding to the conserved SAM-MT motif of “E/DxGxGxG” was identified (Fig. S5). Due to the absence of the conserved motif of “E/D × G × G × G” of SAM-MTs in HtmB1(-module 16)-MT domain (Fig. S6), and we have no clue to do the site-directed mutation of HtmB1(-module 16)-MT domain. At this situation the in-flame deletion of the domain seems reasonable. Thus, in order to establish the direct causal connection between this *O*-methylase domain and methylation at O-21, in frame deletion was conducted to create ΔHtmB1(-module 16)-MT domain. Results showed that the biosynthesis of HTM and relevant TDD and HTM_222_ were also abolished (Fig. S4), but contrast to mutants of CMT(G132D, G134W, G136D) and NTM(G108R, G110R, G112R), even no detectable demethyl-HTM can be found.

### 3.4 Antibacterial bioactivities results

Antibacterial activity results against the eight strains of *M. luteus*, *B. thuringiensis*, *B. subtilis*, *S. aureus*, *A. baumannii*, *K. pneumoniae*, *E. coli*, and *P. aeruginosa* showed that HTM could inhibit the strain of *M*. *luteus*, *B. thuringiensis* and *A. baumannii* with a MIC of 4 μg/mL, 4 μg/mL, and 64 μg/mL, respectively, and no activities against other strains tested in this study at aconcentration of 64 μg/mL (Table 1).

**Table 1:** Antibacterial activities of HTM (MIC, μg/mL).

Compound	*M. luteus*	*B. thuringiensis*	*B. subtilis*	*S. aureus*	*A. baumannii*	*K. pneumoniae*	*E. coli*	*P. aeruginosa*
HTM	4	4	>64	>64	64	>64	>64	>64
Apramycin	16	16	32	1	4	1	2	2
Kanamycin	4	1	8	1	>64	>64	16	>64

## 4 Discussion

In HTM biosynthesis, three methyltransferases existed as the following order of HtmA2(-module 6)-MT domain, HtmB1(-module 16)-MT domain, HtmB2(-module 18)-MT domain. So, we conclude that the *C*-methylation at C-45 will be installed firstly, then the *O*-methylation at O-21, and finally the *N*-methylation at N-12 in HTM with the extension of the HTM PKS/NRPS assembly line. Due to the poor abundance of the intermediates, only one percent of *C*-demethyl-HTM and one in 10000 of *N*-demethyl-HTM was detected in mutants of CMT(G132D, G134W, G136D) and NTM(G108R, G110R, G112R), respectively, however no detectable demethyl-HTM was observed in ΔHtmB1(-module 16)-MT domain. Possibly, it is the difference substrate tolerance of HtmA2(-module 6)-MT domain, HtmB1(-module 16)-MT domain, HtmB2(-module 18)-MT domain in HTM biosynthesis. Although we haven't obtained the intermediates from the mutants, but thanks to cometabolites of HTM_222_ and TDD in HTM biosynthesis containing a *C* and *N*-methyl group that of HTM, respectively, we could detect the demethyl-HTM_222_ and demethyl-TDD in mutants of CMT(G132D, G134W, G136D) and NTM(G108R, G110R, G112R), respectively, and finally determine the action sites of three methyltransferases in HTM. Although, the possibilities that the in-frame deletion may lead to the inactivation of the modular type protein (NRPS) could not be excluded, however, we still could conclude that HtmB1(-module 16)-MT domain is responsible for the installation of *O*-methylation at O-21 according to the results of CMT(G132D, G134W, G136D) and NTM(G108R, G110R, G112R). Through this study, we found that HTM could gradually decreased with time, while the content of HTM_222_ and TDD increased accordingly, and HTM_222_ and TDD may be produced by degradation of HTM. Due to the unique structure and good hepatoprotective bioactivities of HTM, and the abilities of methylation to optimize properties of candidate drugs, the understanding of the methylation in HTM biosynthesis seems very important.

Meanwhile, we reported the antibacterial activities of HTM in this study. HTM was reported previously to have weak antibacterial activities against *Bacillus subtilis* and *Candida albicans* [19], however, in our expanded bioactivities screening, we not only discover its hepatoprotective activities in our previous study [13], but also find its antimicrobial activity against *M*. *luteus*, *B. thuringiensis* and *A. baumannii* with MIC of 4 μg/mL, 4 μg/mL, and 64 μg/mL in this study, respectively, as to the other strains HTM showed no antibacterial activities against to them at a concentration of 64 μg/mL, which are consisted with the antibacterial activities results reported by Zuo et al. [19]. The further deciphering of HTM biosynthetic pathway in this study (methylation in HTM biosynthesis) not only deepen our understanding of HTM's biosynthesis, but also pave the way for the future modifications on the assembly line to generate more HTM derivatives.

In conclusion, we unveil that HtmA2(-module 6)-MT domain, HtmB1(-module 16)-MT domain and HtmB2(-module 18)-MT domain are responsible for the installation of methyl group at C-45, O-21, N-12 of HTM respectively in vivo in this study, and observe that HTM could gradually decrease with time, and HTM_222_ and TDD increase accordingly, suggesting they may be partly produced by degradation of HTM. Further antibacterial activities test unravel that HTM have antibacterial activities against *M*. *luteus*, *B. thuringiensis* and *A. baumannii*. The findings in this study have given us a deeper understanding of HTM's biosynthesis and bioactivities.

## Funding

This work was supported by National Key R&D Program of China (2018YFA0903200), General Program of National Natural Science Foundation of China (32370083), Natural Science Foundation of Chongqing CSTB (CSTB2022NSCQ-MSX0995), and Graduate Innovation Program (GZLCX20232114)

## Declaration of competing interest

The authors declare no conflict of interest.

## CRediT authorship contribution statement

**Minghe Luo:** performed the genetic experiments and LC-ESI-HRMS analysis. **Yulu Dong:** performed the genetic experiments and LC-ESI-HRMS analysis. **Zixin Deng:** analyzed the data and revised the manuscript, is an editorial board member for Synthetic and Systems Biotechnology and was not involved in the editorial review or the decision to publish this article. **Yuhui Sun:** conceived the overall project, analyzed the data and wrote the manuscript.
