# Supplementary material for: Methylation in hangtaimycin biosynthesis and its antibacterial activities
Source: Synth Syst Biotechnol. 2023 Oct 30;8(4):682–7. doi: 10.1016/j.synbio.2023.10.003 (PMC10624959; doi:10.1016/j.synbio.2023.10.003)
Supplement: Multimedia component 1 [file mmc1.docx]

Supplementary Material

Methylation in hangtaimycin biosynthesis and its antibacterial activities

**Minghe Luo ^a, b*^, Yulu Dong ^b^, Zixin Deng ^b^, and Yuhui Sun ^b, *^**

^*^Correspondence: Minghe Luo, [lmh353083@126.com](mailto:lmh353083@126.com), +86-13677697348,

Yuhui Sun, [yhsun@whu.edu.cn](mailto:yhsun@whu.edu.cn), +86-027-68756642;

^a^ School of Pharmacy and Bioengineering, Chongqing University of Technology, Chongqing, China

^b^ Key Laboratory of Combinatorial Biosynthesis and Drug Discovery, Ministry of Education, and School of Pharmaceutical Sciences, Wuhan University, Wuhan, China

Table of contents

**1. Tables**

**Table S1.** Bacterial strains and plasmids used in this study.

Table S2. List of oligonucleotide primers used in this study.

**2. Figures**

**Fig S1.** Alignment of amino acid sequences of methyltransferases in hangtaimycin biosynthesis.

**Fig S2.** Site-directed mutation of HtmA2(-module 6)-MT domain and verification.

**Fig S3.** Site-directed mutation of HtmB2(-module 18)-MT domain and verification.

**Fig S4.** HPLC-DAD analysis at λmax 264 nm of the fermentation extracts of methyltransferase mutants of CMT(G132D, G134W, G136D), NTM(G108R, G110R, G112R), ΔHtmB1(-module 16)-MT domain and wild-type.

**Fig S5.** Alignment of amino acid sequences of FkbM family methyltransferases.

**Fig S6.** Scheme presentation of HtmB1(-module 16)-MT domain deletion and verification.

**Table S1.** Bacterial strains and plasmids used in this study.

| **Strains/Plasmids** | **Characteristics** | **Reference** |
| --- | --- | --- |
| *Micrococcus luteus* | ATCC 10240 (for antibacterial activities test) |  |
| *Bacillus thuringiensis* | BT01 (for antibacterial activities test) |  |
| *Staphylococcus aureus* | ATCC 29213 (for antibacterial activities test) |  |
| *MRSA* | ATCC 43300 (for antibacterial activities test) |  |
| *Acinetobacter baumannii* | ATCC 19606 (for antibacterial activities test) |  |
| *Klebsiella pneumoniae* | ATCC 13883 (for antibacterial activities test) |  |
| *Escherichia coli* | ATCC 25922 (for antibacterial activities test) |  |
| *Pseudomonas aeruginosa* | ATCC 47085 (for antibacterial activities test) |  |
| ***Escherichia coli*** |  |  |
| DH10B | Host for general cloning | Invitrogen |
| ET12567/pUZ8002 | Donor strain for conjugation between *E. coli* and *Streptomyces* | (MacNeil et al., 1992) |
| ***Streptomyces spectabilis*** |  |  |
| CCTCC M2017417 | Wild-type, hangtaimycin producing strain |  |
| CMT(G132D, G134W, G136D) | CMT site-directed mutangenic mutant in HtmA2, hangtaimycin non-producing | This work |
| NTM(G108R, G110R, G112R) | NMT site-directed mutagenic mutant in HtmB2, hangtaimycin non-producing | This work |
| ∆HtmB1(-module 16)-MT domain | HtmOMT in-frame deletion mutant in HtmB1, hangtaimycin non-producing | This work |
| Plasmids |  |  |
| pYH7 Streptomyces | *E. coil*-*Streptomyces* shuttle vector | (Sun et al., 2009) |
| pWHU-CMT | Recombinant construct for CMT site-directed mutation in *htmA2* | This work |
| pWHU-NMT | Recombinant construct for NMT site-directed mutation in *htmB2* | This work |
| pWHU-OMT | Recombinant construct for OMT site-directed mutation in *htmA1* | This work |

Table S2. List of oligonucleotide primers used in this study.

| **Primers** | **Oligonucleotide sequences (5’ to 3’)** | **Restriction site** |
| --- | --- | --- |
| PKS-CMT-L-up | AAGGCGAATACTT**CATATG**CCGTGCTCGTCCTGGACGC | *Nde*I |
| PKS-CMT-L-re | GTCGGTCC**ACGCGT**CGACCTCCAGGACGCGCAGC | *Mlu*I |
| PKS-CMT-R-up | G**ACGCGT**GGACCGACGCGACCAGCGTCAAGGTCC | *Mlu*I |
| PKS-CMT-R-re | GCAGGCATGC**AAGCTT**GGCTCGGCATGCTGTAACG | *Hin*dIII |
| NRPS-NMT-L-up | AAGGCGAATACTT**CATATG**CGACGCCACCCTGTTCAGC | *Nde*I |
| NRPS-NMT-L-re | **ACGCGT**GCGGCAGCGGACCTCCAGGAGCCTGCG | *Mlu*I |
| NRPS-NMT-R-up | CGCTGCCGC**ACGCGT**CTGCTGCTGTTCCGCTACG | *Mlu*I |
| NRPS-NMT-R-re | GCAGGCATGC**AAGCTT**GTGCACGACGGCAGGTCG | *Hin*dIII |
| OMT-L-up | CAAGGCGAATACTT**CATATG**GACGACGTGGTCTTCGGCAC | *Nde*I |
| OMT-L-re | CGAGCTTGCCGGGCAGTTCGTAGGTCCGCT |  |
| OMT-R-up | CGAACTGCCCGGCAAGCTCGACCAGCGGG |  |
| OMT-R-re | ACCTGCAGGCATGC**AAGCTT**CGCGTTCAGCTCCCCGTACG | *Hin*dIII |
| CMT-confirm-up | AGGGCACCGAGATCCTCTTC |  |
| CMT-confirm-re | AGGGTGTTGCGGCTCATCT |  |
| NMT-confirm-up | AGCAGTGGCAGGACCTCTTC |  |
| NMT-confirm-re | AGCCGCTCGATGACCTCTTC |  |
| OMT-comfire-up | CTACGTCCTGGACCCGCACG |  |
| OMT-comfire-re | CGAAGACGGACTGGACGGTGAG |  |

Note: The underlined nucleotides indicate introduced restriction sites.


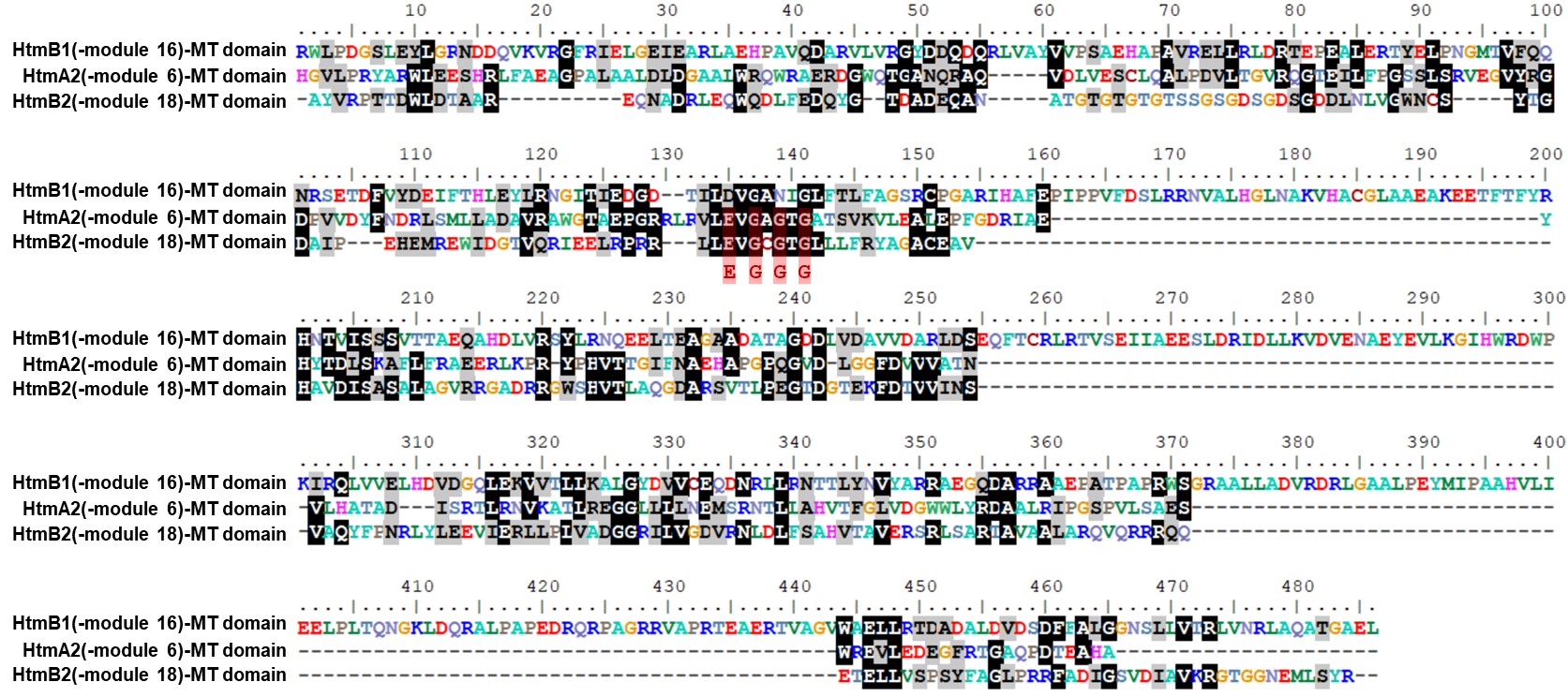


**Fig S1.** Alignment of amino acid sequences of methyltransferases in hangtaimycin biosynthesis. The conserved motif of “E/DxGxGxG” for *S*-adenosylmethionine-dependent methyltransferases in both HtmA2(-module 6)-MT domain and HtmB2(-module 18)-MT domain were marked.


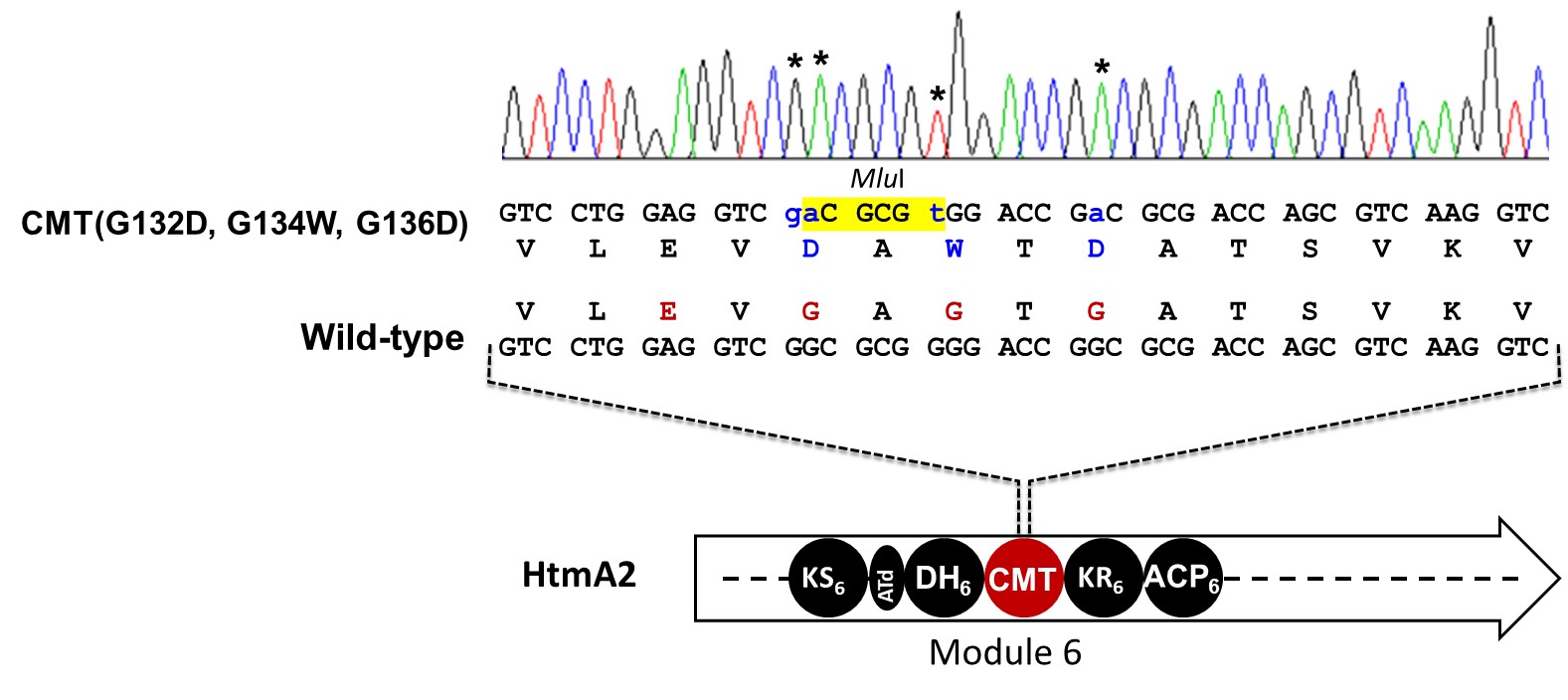


**Fig S2.** Site-directed mutation of HtmA2(-module 6)-MT domain and verification. The conserved SAM-binding motif of “E×G×G×G” in HtmA2(-module 6)-MT domain was mutated to “E×D×W×D”, shown in blue letters and marked with asterisks, resulting in the appearance of a *Mlu*I restriction site which is highlighted with yellow. The mutation was verified by restriction digestion and sequencing.


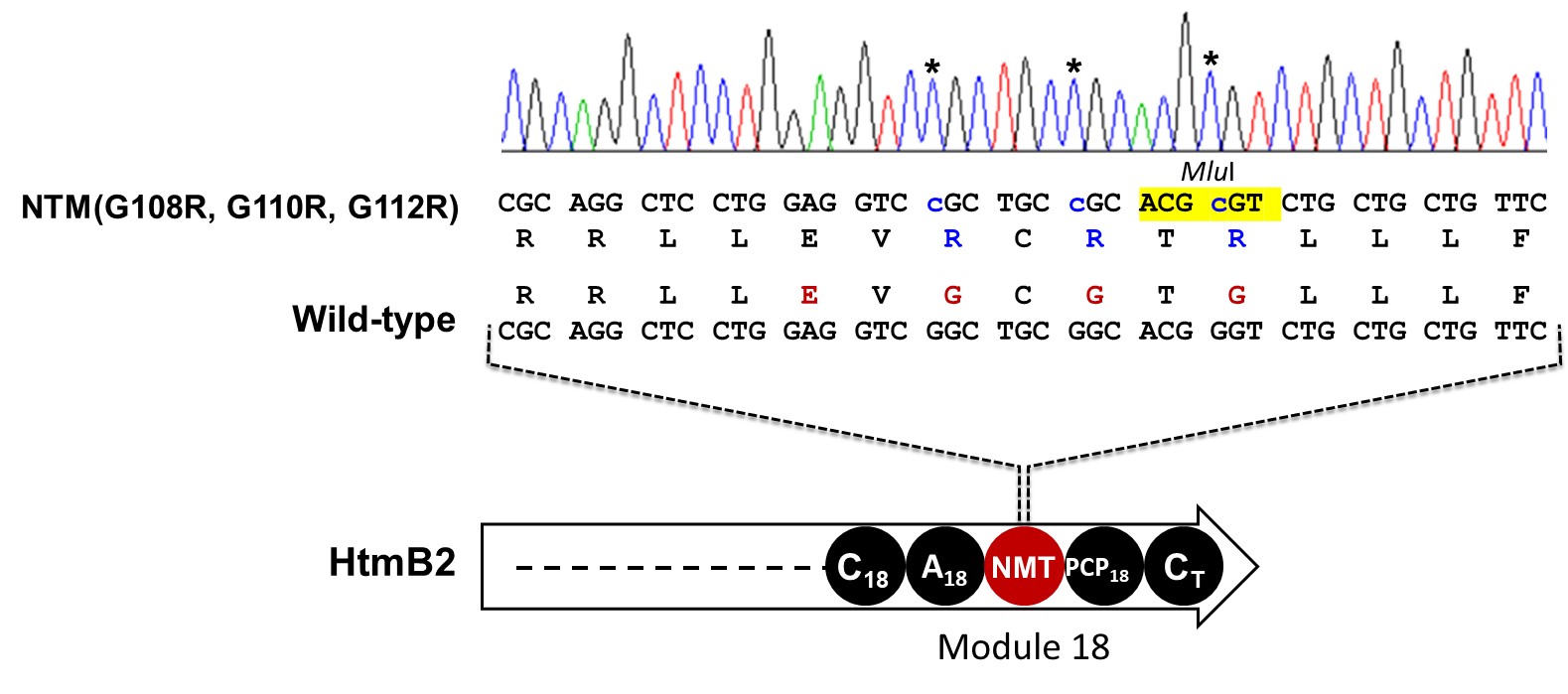


**Fig S3.** Site-directed mutation of HtmB2(-module 18)-MT domain and verification. The conserved SAM-binding motif of “E×G×G×G” in HtmB2(-module 18)-MT domain was mutated to “E×R×R×R”, shown in blue letters and marked with asterisks, resulting in the appearance of a *Mlu*I restriction site which is highlighted with yellow. The mutation was verified by restriction digestion and sequencing.


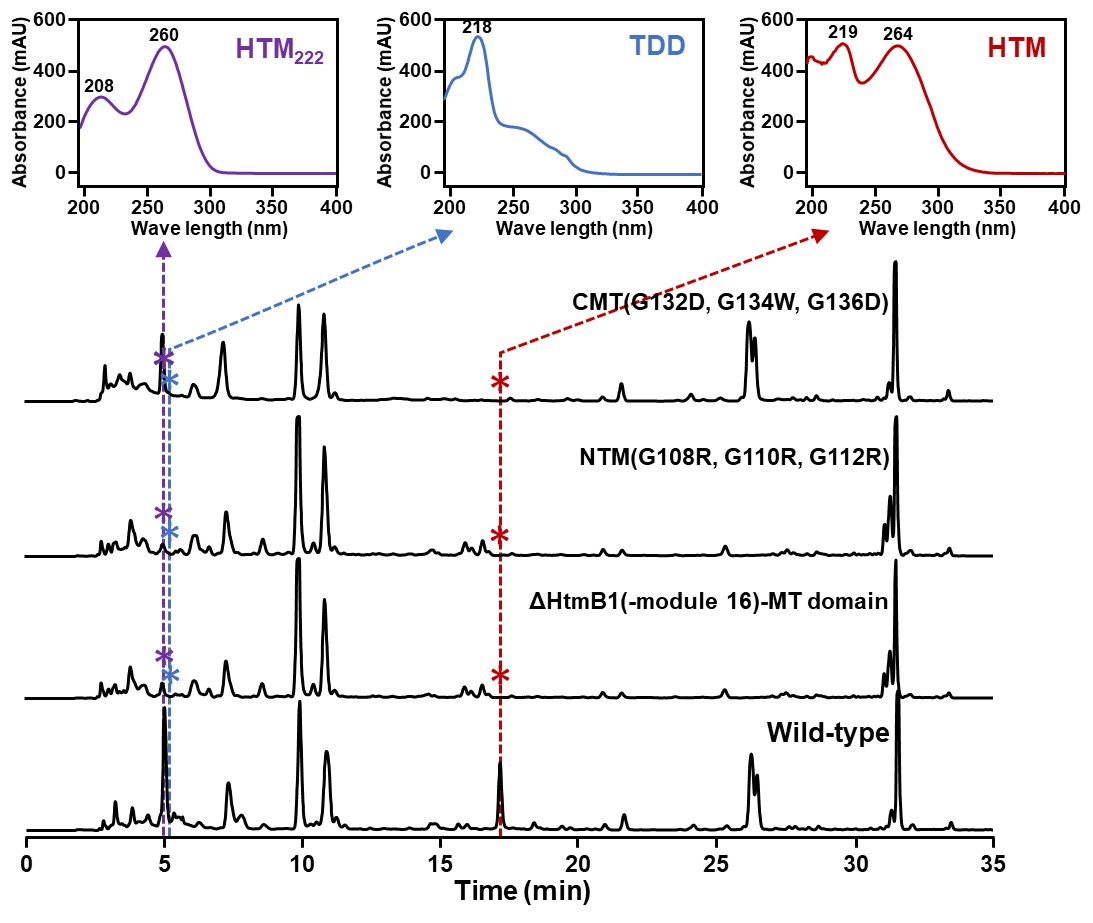


**Fig S4.** HPLC-DAD analysis at λmax 264 nm of the fermentation extracts of methyltransferase mutants CMT(G132D, G134W, G136D), NTM(G108R, G110R, G112R), ΔHtmB1(-module 16)-MT domain and wild-type. The asterisks indicated no corresponding compounds were detected. The UV absorption spectrum of TDD and HTM_222_ obviously differs from HTM.


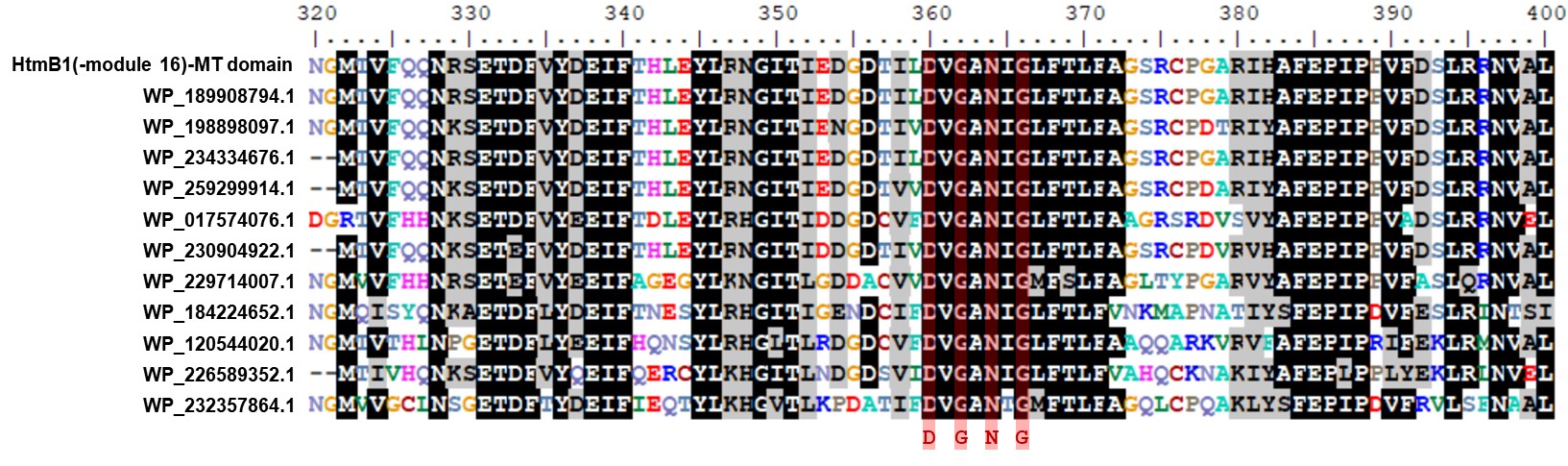


**Fig S5.** Alignment of amino acid sequences of FkbM family methyltransferases. The conserved motif of “DxGxNxG” corresponding to conserved motif of “E/DxGxGxG” for *S*-adenosylmethionine-dependent methyltransferases were marked. WP_189908794.1 from *Streptomyces capillispiralis*; WP_198898097.1from *Streptomyces flavofungini*; WP_234334676.1from *Streptomyces* sp. NRRL B-1347; WP_259299914.1from *Streptomyces aurantiacus*; WP_017574076.1from *Nocardiopsis halotolerans*; WP_184224652.1from *Granulicella aggregans*; WP_120544020.1from *Corallococcus terminator*; WP_226589352.1from *Microseira wollei*; WP_232357864.1from *Paraneptunicella aestuarii*.


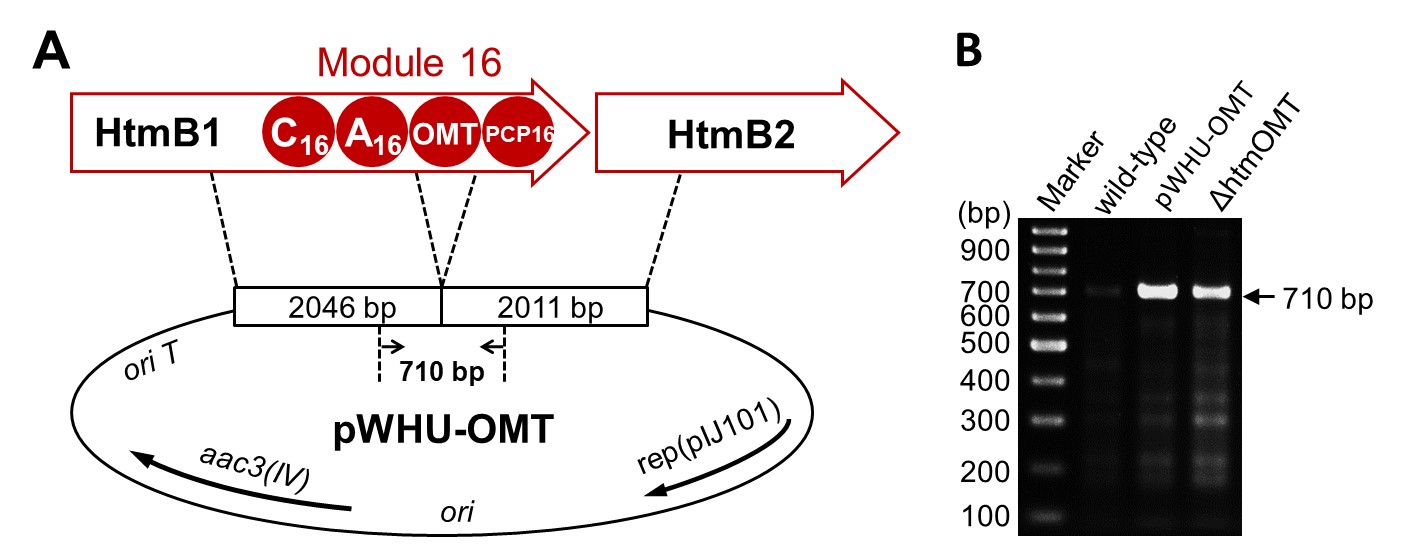


**Fig S6.** Scheme presentation of HtmB1(-module 16)-MT domain deletion and verification. (A) In-frame deletion of module 16 mediated by double homologous recombination in vivo. (B) The mutant is confirmed by PCR amplification and sequencing using the genomic DNA from the mutant of ΔHtmB1(-module 16)-MT domain, wild-type strain (negative control) and plasmid pWHU-OMT (positive control) as the template, respectively.

# Reference

Macneil DJ, Occi JL, Gewain KM, Macneil T, Gibbons PH, Ruby CL, and Danis SJ (1992) Complex organization of the *Streptomyces avermitilis* genes encoding the avermectin polyketide synthase. Gene 115:119-125. doi: 10.1016/0378-1119(92)90549-5

Sun Y, He X, Liang J, Zhou X, and Deng Z (2009) Analysis of functions in plasmid pHZ1358 influencing its genetic and structural stability in *Streptomyces* lividans 1326. Appl Microbiol Biotechnol 82:303-310. doi: 10.1007/s00253-008-1793-7
